# Macrophages Control the Bioavailability of Vitamin D and Vitamin D-Regulated T Cell Responses

**DOI:** 10.3389/fimmu.2021.722806

**Published:** 2021-09-21

**Authors:** Daniel Villalba Lopez, Fatima A. H. Al-Jaberi, Anders Woetmann, Niels Ødum, Charlotte Menné Bonefeld, Martin Kongsbak-Wismann, Carsten Geisler

**Affiliations:** The LEO Foundation Skin Immunology Research Center, Department of Immunology and Microbiology, Faculty of Health and Medical Sciences, University of Copenhagen, Copenhagen, Denmark

**Keywords:** Vitamin D, DBP, macrophages, T cells, cytokines

## Abstract

The active form of vitamin D_3_ (1,25(OH)_2_D_3_) has a great impact on T cell effector function. Thus, 1,25(OH)_2_D_3_ promotes T helper 2 (Th2) and regulatory T (Treg) cell function and concomitantly inhibits Th1 and Th17 cell function. Thus, it is believed that vitamin D exerts anti-inflammatory effects. However, vitamin D binding protein (DBP) strongly binds both 1,25(OH)_2_D_3_ and the precursor 25(OH)D_3_, leaving only a minor fraction of vitamin D in the free, bioavailable form. Accordingly, DBP in physiological concentrations would be expected to block the effect of vitamin D on T cells and dendritic cells. In the present study, we show that pro-inflammatory, monocyte-derived M1 macrophages express very high levels of the 25(OH)D-1α-hydroxylase CYP27B1 that enables them to convert 25(OH)D_3_ into 1,25(OH)_2_D_3_ even in the presence of physiological concentrations of DBP. Co-cultivation of M1 macrophages with T cells allows them to overcome the sequestering of 25(OH)D_3_ by DBP and to produce sufficient levels of 1,25(OH)_2_D_3_ to affect T cell effector function. This study suggests that in highly inflammatory conditions, M1 macrophages can produce sufficient levels of 1,25(OH)_2_D_3_ to modify T cell responses and thereby reduce T cell-mediated inflammation *via* a vitamin D-mediated negative feed-back loop.

## Introduction

Upon antigen recognition, naïve CD4^+^ T helper (Th) cells become activated and differentiate into various T cell subsets defined by the lineage-specific master transcription factors they express and the cytokines they produce (1). Several studies have demonstrated that the active form of vitamin D, 1,25(OH)_2_D_3_, modulates the effector function of Th cells *in vitro*. Thus, 1,25(OH)_2_D_3_ promotes Th2 and Treg cell effector function by increasing expression of the transcription factors GATA3 and FoxP3 and the production of IL-4, IL-5, IL-13 and IL-10 (2–11), while it concomitantly inhibits Th1 and Th17 cell effector function by reducing expression of Tbx21 and RORγt and the production of IFN-γ and IL-17A (5–9, 12–18). Thus, it is believed that vitamin D exerts anti-inflammatory roles during immune responses *in vivo*, which is in line with the observations that vitamin D deficiency is associated with increased risk of autoimmune disorders such as lupus erythematous, multiple sclerosis and type I diabetes mellitus (19–21). The inactive precursor of 1,25(OH)_2_D_3_ is 25-hydroxyvitamin D_3_ (25(OH)D_3_) and its serum concentration is regarded as the best clinical indicator for the vitamin D status of a subject (22). Serum concentrations of 25(OH)D_3_ is normally between 50 and 125 nM, whereas the serum concentration of 1,25(OH)_2_D_3_ is approximately 1000-fold lower being between 60-110 pM. It is estimated that more than 99% of 25(OH)D_3_ and 1,25(OH)_2_D_3_ is bound to the vitamin D binding protein (DBP) and albumin with less than 1% in the free form (23, 24). By use of a mathematical model, it was recently predicted that at 50 nM 25(OH)D_3_ and 100 pM 1,25(OH)_2_D_3_ only 0.1% of 25(OH)D_3_ and 1.5% of 1,25(OH)_2_D_3_ were in the free form *in vivo* (25). Traditionally, the function of converting 25(OH)D_3_ into 1,25(OH)_2_D_3_ has been ascribed to the proximal tubular cells of the kidneys due to their expression of the DBP transporters megalin and cubulin and the 25(OH)D_3_-1α-hydroxylase CYP27B1 (26, 27). Interestingly, immune cells, such as T cells and monocyte-derived cells, also express CYP27B1, which in theory allows them to produce 1,25(OH)_2_D_3_ (11, 28, 29). However, these cells do not have the ability to endocytose DBP *via* megalin/cubulin, meaning that only the very limited fraction of the free form of 25(OH)D_3_ is available for immune cells for the conversion to 1,25(OH)_2_D_3_ (10, 30). We and others have shown that activated CD4^+^ T cells express CYP27B1 in sufficient high concentrations to convert 25(OH)D_3_ to 1,25(OH)_2_D_3_ *in vitro* (10, 11, 31). Likewise, monocytes and monocyte-derived dendritic cells express CYP27B1 and can convert 25(OH)D_3_ to 1,25(OH)_2_D_3_ *in vitro* (30, 32). Importantly, addition of DBP even in concentrations below the physiological concentration of ∼5 µM abolished the conversion of 25(OH)D_3_ to 1,25(OH)_2_D_3_ and the impact of 25(OH)D_3_ on T cell responses. These studies suggested that the presence of DPB *in vivo* excludes the possibility for immune cells to convert 25(OH)D_3_ to 1,25(OH)_2_D_3_ and thereby to be affected by vitamin D. However, several observations indicate that 1,25(OH)_2_D_3_ can be generated *in vivo* even in high amounts during some types of immune responses involving granulomas containing high numbers of activated macrophages and T cells. This has been described in patients with granulomatous disorders, most commonly in sarcoidosis (33–35) and tuberculosis (36), and it results in a condition characterized by normal 25(OH)D_3_, elevated 1,25(OH)_2_D_3_, hypercalcemia and hypercalciuria that ultimately may lead to renal failure.

The primary cellular components of granulomas are macrophages and T cells. Macrophages can be broadly classified in two main groups, namely M1 macrophages that exhibit potent microbicidal properties and promote strong IL-12-mediated Th1 responses, and M2 macrophages that support Th2 responses (37). Granulomas can be dominated by both M1 and M2 macrophages (38). It has been suggested that macrophages express more CYP27B1 than T cells (28) and that the expression levels of CYP27B1 is detrimental for 1,25(OH)_2_D_3_ production *in vivo* (32). However, the expression levels of CYP27B1 in M1 and M2 macrophages and their capability to convert 25(OH)D_3_ to 1,25(OH)_2_D_3_ in the presence of DBP remain to be determined.

## Materials and Methods

### Reagents and Chemicals

25(OH)D_3_ (Cat: BML-DM-100-0001) was from Enzo Life Sciences, Inc., Ann Arbor, MI. 25(OH)D_3_ stock solution of 2.5 mM was prepared in > 99.5% anhydrous ethanol. DBP (Cat: A50674H) was from Meridian Life Science and diluted in sterile PBS. TNFα (Cat: 210-TA), IL-1β (Cat: 201-LB), IL-6 (Cat: 206-IL) and IL-23 (Cat: 1290-IL) were purchased from R&D systems. Galunisertib (Cat: LY2157299) was purchased from Selleckchem and the AhR agonist FICZ (Cat: 5304) was from TOCRIS Inc.

### Monocyte-Derived M1 and M2 Macrophages

All procedures involving the handling of human samples in this study were in accordance with the principles described in the Declaration of Helsinki and the samples were collected and analysed according to ethically approval by the Regional Ethical Committee of the Capital Region of Denmark (H-16033682). Human monocytes were purified from PBMC using Easysep Human Monocyte Enrichment Kit (Cat:19059 Stemcell Technologies) according to the manufacturer’s protocol. To generate M1 macrophages, 1.5 x 10^6^ monocytes were cultured in flat-bottomed 6-well culture plates (Cat: 140675, Nunc) for six days in 3 ml of M1 medium (RPMI-1640 medium (Cat: R5886, Sigma Aldrich) supplemented with 1% Penicillin/Streptomycin, 1% L-Glutamine, 10% heat-inactivated, endotoxin-free fetal bovine serum (FBS) (Cat: 10082-147, Gibco) and GM-CSF (50 ng/ml, Cat: AF-HDC, Peprotech)). To generate M2 macrophages, 1.5 x 10^6^ monocytes were cultured in flat-bottomed 6-well culture plates (Cat: 140675, Nunc) for six days in 3 ml of M2 medium (RPMI-1640 medium (Cat: R5886, Sigma Aldrich) supplemented with 1% Penicillin/Streptomycin, 1% L-Glutamine, 10% heat-inactivated, endotoxin-free FBS, GM-CSF and IL-4 (both 50 ng/ml, Cat: AF-HDC, Peprotech)). After three days, fresh M1 and M2 medium was added to the cultures. On day five, M1 macrophages were activated with IFNγ (50 ng/ml) (Cat: 285-IF-100/CF, R&D Systems) and LPS (50 ng/ml) (Cat: 5568, Sigma-Aldrich) and M2 macrophages were activated with LPS (50 ng/ml) (Cat: 5568, Sigma-Aldrich) for 24 hours. At day 6, activated M1 and M2 macrophages were washed in PBS and resuspended in X-VIVO 15 for mono- and co-cultures. For mono-culture, 5 x 10^5^ of M1 or M2 macrophages per ml were cultured in flat-bottomed, 24-well plates for 0-120 h in the presence of the indicated concentrations of 25(OH)D_3_ and DBP. For co-culture experiments, 1 x 10^6^ naïve human CD4^+^ T cells were co-cultured with 1 x 10^5^ allogeneic M1 or M2 macrophages per ml in flat-bottomed, 24-well culture plates in X-VIVO 15 medium in the presence of the indicated concentrations of 25(OH)D_3_ and DBP.

### T Cell Isolation and Activation

Peripheral blood mononuclear cells (PBMC) were purified from the blood of healthy donors by density gradient centrifugation using Lymphoprep (Axis-Shield, Oslo, Norway). Subsequently, naive CD4^+^ T cells were isolated by negative selection using Easysep Human Naive CD4^+^ T cell Enrichment Kit (Cat:19155 Stemcell Technologies) according to the manufacturer’s protocol. In short, PBMC were incubated with antibodies targeting undesired cells, and subsequently magnetic particles were used to bind desired cells. Hereafter, these cells were retained using an EasySep Magnet (Cat:18000, Stemcell Technologies). The resulting cell population consisted of > 95% naïve CD4^+^ T cells (39). The obtained cells were cultured at a concentration of 1 x 10^6^ cells/ml serum-free X-VIVO 15 medium (Cat: BE02-060F, Lonza, Verviers, Belgium) and activated with macrophages in a 1:10 macrophage:T cell ratio or with Dynabeads Human T-activator CD3/CD28 (Cat:111.31D, Life Technologies, Grand Island, NY) in a 2:5 bead:T cell ratio for 96 hours in flat-bottomed 24 well culture plates (Cat:142475, Nunc).

### RT-qPCR

mRNA levels for various targets were measured by RT-qPCR. Isolated cells were lysed in TRI reagent (Cat: T9424, Sigma Aldrich) and mixed with 1-bromo-3-chloropropane (BCP) (Cat: B9673, Sigma Aldrich). The aqueous phase containing the RNA sample was precipitated using isopropanol supplemented with glycogen (Cat: 10814-010, Invitrogen), washed with ethanol and dissolved in RNase free water. Next, the synthesis of cDNA was performed with equal amounts of total RNA using the High-Capacity RNA-to-cDNA™ Kit from Applied Biosystems (Cat: 4387406) according to manufacturer’s protocol. For RT-qPCR, 12.5 ng of cDNA was mixed with TaqMan^®^ Universal Master Mix II with UNG (Cat: 4440038, Applied Biosystems), the control eukaryotic 18 S rRNA primer (Cat: 1509311, Applied Biosystems), the target primer and RNase/DNase free water for normalization. The following target primers were used: VEGFA (Hs00900055_m1), CCR7 (Hs01013469_m1), CD80 (Hs01045161_m1), IL-1β (Hs00907314_m1), MRC1 (Hs07288635_g1), PDGFB (Hs00966522_m1), TIMP3 (Hs00165949_m1), IL-6 (Hs00985639_m1), TNF (Hs01113624_g1), IL-12A (Hs01073447_m1), CYP2R1 (Hs01379776_m1), CYP27A1 (Hs01017992_m1), CYP27B1 (Hs01096154_m1), Tbx21 (Hs00203436_m), GATA3 (Hs00231122_m1), FoxP3 (Hs01085834_m1) and RORC (Hs01076122_m1) all from Applied Biosystems. The samples were run on a LightCycler ^®^ 480 II from Roche for real-time PCR amplification.

### Cytokine Measurements

IFNγ, IL-4, -5, -10, -13 and -17A were measured by ELISA according to the manufacturer´s instruction (IFNγ (Cat: 88-7316-88), IL-4 (Cat: 88-7046-88), IL-5 (Cat: 88-7056-88), IL-13 Cat: 88-7439-88 and IL-17A (Cat: 88-7176-88) all from ThermoFisher Scientific).

### 1,25(OH)_2_D_3_ Measurements

To determine the ability of the macrophages and T cells to convert 25(OH)D_3_ into 1,25(OH)_2_D_3_ in mono- and co-cultures, the concentration of 1,25(OH)_2_D_3_ in the supernatants of cells cultured in the presence of 25(OH)D_3_ was measured using the 1,25-Dihydroxy Vitamin D EIA kit (Cat: AC-62F1, Immunodiagnostics Systems) as previously described (11). In short, supernatants were incubated on immunocapsules containing anti-1,25(OH)_2_D_3_ in order to extract 1,25(OH)_2_D_3_. Following extraction, 1,25(OH)_2_D_3_ was eluded into borosilicate tubes, and the elution reagent was evaporated under gentle flow of liquid nitrogen. 1,25(OH)_2_D_3_ was subsequently resuspended in buffer and concentrations were determined by a competitive ELISA with biotin-coupled 1,25(OH)_2_D_3_.

### Statistical Analysis

Two-tailed, paired Student’s t-tests were used to compare responses in the same group of cells treated in two different ways. In other cases, one- or two-way ANOVA tests were used as indicated in the figure legends. Significance levels are indicated for the adjusted p-values as *^,#,¤,$^ p < 0.05; **^,##,¤¤,$$^ p < 0.01; ***^,###,¤¤¤,$$$^ p < 0.005; ****^,####,¤¤¤¤,$$$$^ p < 0.001. Data are presented as mean values with one standard error of the mean (SEM). The number of donors and the number of independent experiments are indicated in the figure legends.

## Results

### Differentiation of Human Monocyte-Derived M1 and M2 Macrophages *In Vitro*

**Figure 1:**
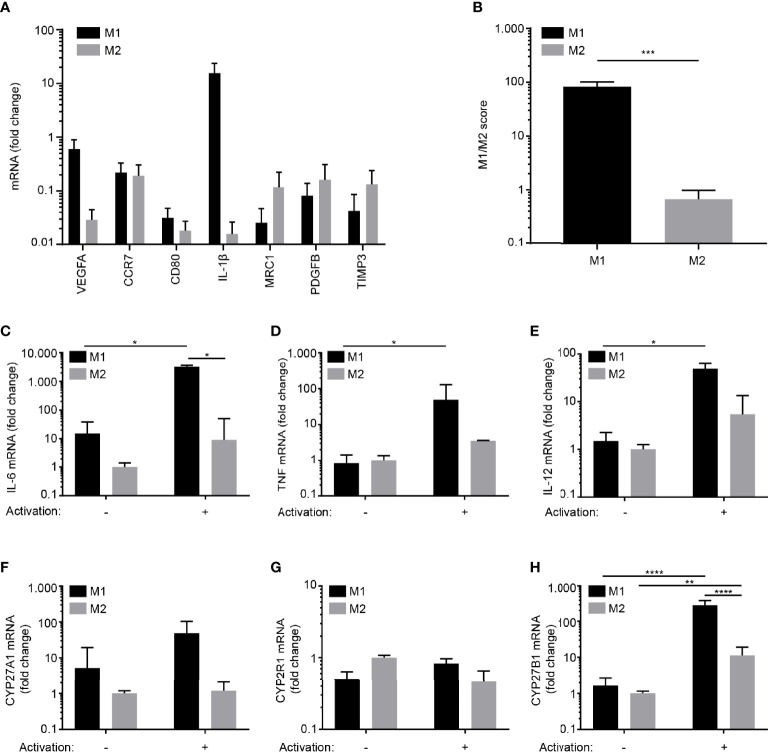
Differentiation of human monocyte-derived M1 and M2 macrophages *in vitro*. **(A)** Relative mRNA expression of M1 (VEGF, CCR7, CD80 and IL-1β) and M2 signature markers (MRC1, PDGFB and TIMP3) in M1 and M2 macrophages activated with LPS and IFNγ and LPS, respectively. **(B)** M1/M2 score in activated M1 and M2 macrophages. Data (mean + SEM) were obtained from two independent experiments with four donors and tested by two-tailed, paired Student’s t-tests. Relative mRNA expression of **(C)** IL-6, **(D)** TNF, **(E)** IL-12A, **(F)** CYP27A1 **(G)** CYP2R1and **(H)** CYP27B1 in resting and activated M1 and M2 macrophages. The data in **(C–H)** are normalized to the values obtained from non-stimulated M2 macrophages. Data (mean + SEM) were obtained from three independent experiments with five donors and tested by one-way ANOVA with *post hoc* multiple comparisons test (Tukey’s).

We differentiated monocytes towards M1 macrophages with GM-CSF followed by activation with LPS and IFNγ and towards M2 macrophages with GM-CSF and IL-4 followed by activation with LPS as previously described (40, 41). We subsequently measured the mRNA expression levels of the four pro-inflammatory genes *VEGF*, *CCR7*, *CD80* and *IL1B* and the three anti-inflammatory genes *MRC1*, *PDGFB* and *TIMP3* that have been identified to be differentially expressed in M1 and M2 macrophages (40, 41). In line with previous studies, we found that the pro- and anti-inflammatory genes were higher expressed in M1 and M2 macrophages, respectively (**Figure 1A**). This was also reflected in the M1/M2 score as defined in (41) (**Figure 1B**). We next determined the expression of pro-inflammatory cytokines and enzymes involved in 1,25(OH)_2_D_3_ synthesis in resting and activated M1 and M2 macrophages. We did not find any significant differences in the expression levels of the selected genes in resting M1 and M2 macrophages (**Figures 1C–H**). In contrast, activated M1 macrophages expressed significantly more IL-6 and most importantly CYP27B1 than activated M2 macrophages (**Figures 1C–H**). Taken together, these data demonstrated that activated, pro-inflammatory M1 macrophages express more CYP27B1 than activated M2 macrophages, indicating that M1 macrophages might have the highest capacity to convert 25(OH)D_3_ to 1,25(OH)_2_D_3_.

### M1 Macrophages Strongly Up-Regulate CYP27B1 and Produce More 1,25(OH)_2_D_3_ Than M2 Macrophages and CD4^+^ T Cells

**Figure 2:**
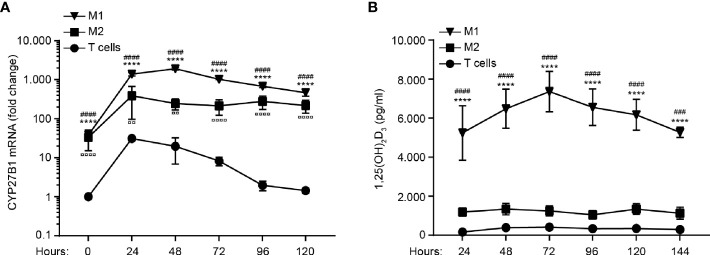
M1 macrophages strongly up-regulate CYP27B1 and produce more 1,25(OH)_2_D_3_ than M2 macrophages and CD4^+^ T cells. Relative mRNA expression of CYP27B1 **(A)** and production of 1,25(OH)_2_D_3_ **(B)** in M1 and M2 macrophages and CD4^+^ T cells activated with LPS and IFNγ, LPS and Dynabeads Human T-activator CD3/CD28, respectively, for 0 to 120 hours in the presence of 100 nM 25(OH)D_3_. Data in **(A)** are normalized to CYP27B1 in CD4^+^ T cells at time zero and were obtained from three independent experiments with nine donors (3 T cell donors, 3 M1 macrophage donors and 3 M2 macrophage donors). Data in **(B)** were obtained from three independent experiments with 17 donors (6 T cell donors, 6 M1 macrophage donors and 5 M2 macrophage donors). *M1 macrophages *versus* T cells; ^#^M1 macrophages *versus* M2 macrophages; ^¤^ M2 macrophages *versus* T cells. **(A, B)** Data (mean ± SEM) were tested by use of two-way ANOVA with *post hoc* multiple comparisons test (Tukey’s).

To determine the capacity to convert 25(OH)D_3_ to 1,25(OH)_2_D_3_, we activated M1 and M2 macrophages and naïve CD4^+^ T cells for 0 – 120 hours in the presence of 25(OH)D_3_ and measured CYP27B1 mRNA levels and the production of 1,25(OH)_2_D_3_ in parallel. We found that M1 macrophages up-regulated CYP27B1 more quickly and strongly than M2 macrophages and T cells (**Figure 2A**). In line with the stronger CYP27B1 expression in M1 macrophages, we found a much higher production of 1,25(OH)_2_D_3_ in M1 macrophages compared to M2 macrophages and CD4^+^ T cells (**Figure 2B**).

### T Cells Enhance the Production of 1,25(OH)_2_D_3_ in M1 and M2 Macrophages

**Figure 3:**
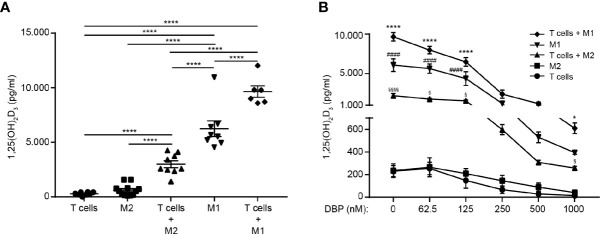
T cells enhance the production of 1,25(OH)_2_D_3_ in M1 and M2 macrophages. **(A)** Production of 1,25(OH)_2_D_3_ in mono-cultures of activated CD4^+^ T cells, M1 and M2 macrophages and in co-cultures of CD4^+^ T cells and M1 and M2 macrophages cultured in the presence of 100 nM 25(OH)D_3_ for 96 hours. Data (mean ± SEM) were obtained from five independent experiments with 12 donors (8 T cells donors 6 M1 macrophage donors, 12 M2 macrophage donors, 6 donor pairs for T cells in co-culture with M1 macrophages (2 T cell donors and 3 M1 macrophage donors mixed to 6 combinations) and 9 donor pairs for T cells in co-culture with M2 macrophages (3 T cell donors and 3 M2 macrophage donors mixed to 9 combinations) and were tested by use of one-way ANOVA with *post hoc* multiple comparisons test (Tukey’s). **(B)** Production of 1,25(OH)_2_D_3_ in mono-cultures of activated CD4^+^ T cells, M1 and M2 macrophages and in co-cultures of CD4^+^ T cells and M1 and M2 macrophages cultured in the presence of 100 nM 25(OH)D_3_ and DBP at the indicated concentrations for 96 hours. Data (mean ± SEM) were obtained from three independent experiments with 8 donors (7 T cells donors 3 M1 macrophage donors, 6 M2 macrophage donors, 6 donor pairs for T cells in co-culture with M1 macrophages (2 T cell donors and 3 M1 macrophage donors mixed to 6 combinations) and 4 donor pairs for T cells in co-culture with M2 macrophages (2 T cell donors and 2 M2 macrophage donors mixed to 4 combinations) and were tested by use of two-way ANOVA with *post hoc* multiple comparisons test (Tukey’s). *T cells + M1 macrophages *versus* M1 macrophages; ^#^M1 macrophages *versus* T cells + M2 macrophages; ^$^T cells + M2 macrophages *versus* M2 macrophages.

Previous studies have demonstrated that activated T cells can enhance the expression of CYP27B1 in monocytes, dendritic cells and macrophages (30, 42). To determine whether T cells have the ability to enhance CYP27B1 and thereby the conversion of 25(OH)D_3_ to 1,25(OH)_2_D_3_ in M1 and M2 macrophages, we compared the production of 1,25(OH)_2_D_3_ in mono-cultures of activated M1 macrophages, M2 macrophages and T cells with co-cultures of T cells with activated, allogeneic M1 or M2 macrophages. We cultured the cells for 96 hours in the presence of 25(OH)D_3_ and subsequently measured the concentration of 1,25(OH)_2_D_3_ in the supernatants. We found that T cells significantly increased the production of 1,25(OH)_2_D_3_ in the supernatants of co-cultures with M1 and M2 macrophages (**Figure 3A**).

Previous studies found that DBP in sub-clinical concentrations completely inhibited the conversion of 25(OH)D_3_ to 1,25(OH)_2_D_3_ in T cells and dendritic cells (10, 30). To determine whether the strong expression of CYP27B1 in M1 macrophages was sufficient to allow for an effective conversion of 25(OH)D_3_ to 1,25(OH)_2_D_3_ even in the presence of DBP, we compared the production of 1,25(OH)_2_D_3_ in activated M1 macrophages, M2 macrophages and T cells in mono- and co-cultures. We cultured the cells for 96 h in the presence of 25(OH)D_3_ and increasing concentrations of DBP and subsequently measured the concentration of 1,25(OH)_2_D_3_ in the supernatants. As previously demonstrated, we found that DBP in physiological concentrations completely abolished 1,25(OH)_2_D_3_ production in isolated T cells and M2 macrophages. However, M2 macrophages in co-culture with T cells, M1 macrophages in mono-cultures and, most obvious, M1 macrophages in co-culture with T cells, produced significant amounts of 1,25(OH)_2_D_3_ even in the highest concentration of DBP tested (**Figure 3B**).

### Co-Cultures of M1 Macrophages and T Cells Produce Sufficient 1,25(OH)_2_D_3_ to Affect T Cell Effector Function in the Presence of High Concentrations of DBP

**Figure 4:**
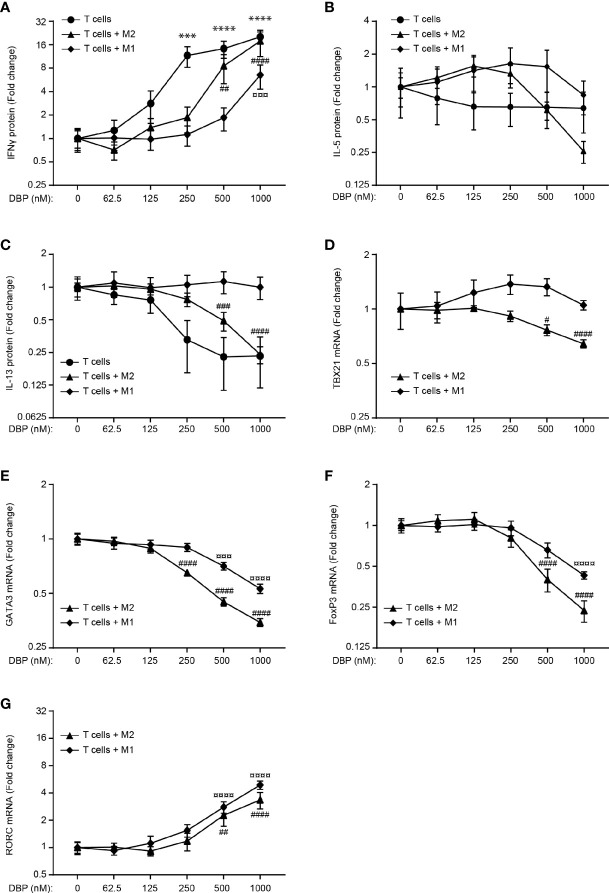
Co-cultures of M1 macrophages and T cells produce sufficient 1,25(OH)_2_D_3_ to affect T cell effector function in the presence of high concentrations of DBP. Relative **(A)** IFN-γ, **(B)** IL-5 and **(C)** IL-13 concentrations in the supernatant of CD4^+^ T cells activated in mono-cultures with Dynabeads Human T-activator CD3/CD28 and CD4^+^ T cells co-cultured with allogeneic M1 and M2 macrophages activated with LPS and IFNγ and LPS, respectively, for 96 hours in the presence of 100 nM 25(OH)D_3_ and DBP at the indicated concentrations. Each series of data was normalized to the cytokine production in the presence of 100 nM 25(OH)D_3_ and absence of DBP. Relative expression of Tbx21 **(D)**, GATA3 **(E)**, FoxP3 **(F)** and RORC **(G)** in CD4^+^ T cells co-cultured with allogeneic M1 and M2 macrophages activated with LPS and IFNγ and LPS, respectively, for 96 hours in the presence of 100 nM 25(OH)D_3_ and DBP at the indicated concentrations. Data (mean ± SEM) were obtained from two to five independent experiments with 6 donors (4 T cells donors, 8 donor pairs for T cells in co-culture with M1 macrophages (4 T cell donors and 2 M1 macrophage donors mixed to 8 combinations) and 6 donor pairs for T cells in co-culture with M2 macrophages (3 T cell donors and 2 M2 macrophage donors mixed to 6 combinations). The data sets were tested using a one-way ANOVA with *post hoc* multiple comparisons test (Dunnett’s) to the cell cultures without DBP. *T cells treated with DBP *versus* T cells without DBP; ^#^T cells + M2 macrophages treated with DBP *versus* T cells + M2 macrophages without DBP; ^¤^T cells + M1 macrophages treated with DBP *versus* T cells + M1 macrophages without DBP.

To determine whether M1 and M2 macrophages had the capability to produce sufficient amounts of 1,25(OH)_2_D_3_ to affect T cell effector function in the presence of DBP, we activated T cells with Dynabeads Human T-activator CD3/CD28 in mono-cultures or with activated, allogeneic M1 or M2 macrophages for 96 hours in the presence of 25(OH)D_3_ and increasing concentrations of DBP and subsequently measured IFNγ, IL-4, -5, -10, -13 and -17A in the supernatants by ELISA. We found that DBP in the highest concentration tested abolished the effect of 25(OH)D_3_ on IFNγ (**Figure 4A**), IL-5 (**Figure 4B**) and IL-13 (**Figure 4C**) in T cells in mono-cultures and in co-cultures with M2 macrophages. However, in co-cultures of T cells and M1 macrophages, sufficient 1,25(OH)_2_D_3_ was produce to allow for 1,25(OH)_2_D_3_-mediated inhibition of IFN-γ and stimulation of IL-5 and IL-13 production even in the highest concentration of DBP tested (**Figures 4A–C**). We could not detect IL-4 above the detection limit in any of the cultures. Likewise, we only detected IL-10 and IL-17A above the detection limit in T cells in mono-culture but not in co-cultures with M1 or M2 macrophages (**Supplementary Figure 1**).

These data supported that more of the active form of vitamin D was produced in T cell/M1 macrophage co-cultures than in T cell/M2 macrophage co-cultures in the presence of DBP as shown in **Figure 3**. To further analyse whether M1 and M2 macrophages had the capability to produce sufficient amounts of 1,25(OH)_2_D_3_ to affect the expression of central transcription factors in the presence of DBP, we activated T cells with activated, allogeneic M1 or M2 macrophages for 96 hours in the presence of 25(OH)D_3_ and increasing concentrations of DBP and subsequently measured the expression of Tbx21, GATA3, FoxP3 and RORC by RT-qPCR. As seen for the cytokines, we found that vitamin D-regulated expression of Tbx21, GATA3 and FoxP3 was more resistant to DBP in T cell/M1 macrophage co-cultures than in T cell/M2 macrophage co-cultures (**Figures 4D–F**). In line with the non-detectable IL-17A production in T cells in co-culture with M1 and M2 macrophages, RORC was only weakly expressed and regulated by 1,25(OH)_2_D_3_ in these cultures (**Figure 4G**). Taken together, these data supported that co-cultures of T cells and M1 macrophages produced sufficient amounts of 1,25(OH)_2_D_3_ to affect T cell effector function in the presence of high concentrations of DBP.

## Discussion

In this study, we demonstrate that activated pro-inflammatory M1 macrophages express higher levels of CYP27B1 than M2 macrophages and T cells. This was reflected by the superior ability of activated M1 macrophages to convert 25(OH)D_3_ to 1,25(OH)_2_D_3_. In addition, we show that T cells could further enhance the ability of activated M1 and M2 macrophages to convert 25(OH)D_3_ to 1,25(OH)_2_D_3_. This is in accordance with previous studies, which found that activated T cells augmented the expression of CYP27B1 in monocytes, dendritic cells and macrophages (30, 42). Interestingly, we found that resting M1 and M2 macrophages expressed similar levels of CYP27B1, but already 24 h after activation with LPS and IFN-γ, M1 macrophages expressed 5-10 fold more CYP27B1 and produced 5 times more 1,25(OH)_2_D_3_ than M2 macrophages activated with LPS alone. This suggested that IFN-γ played an important role in the induction of CYP27B1 in M1 macrophages. Whether IFN-γ or other cytokines or alternative forms of activation could induce similar high levels of CYP27B1 in M2 macrophages as in M1 macrophages would be relevant to determine in future studies.

DBP plays a key role in the bioavailability of 25(OH)D_3_ and 1,25(OH)_2_D_3_, and it has been calculated that only 0.1% of 25(OH)D_3_ and 1.5% of 1,25(OH)_2_D_3_ are in the free form *in vivo* (25). Mathematical modelling has predicted that 25(OH)D_3_ even at 100 nM would have no effect on monocytes *in vivo* due to the presence of DBP. Only by increasing the expression of CYP27B1 by a factor 10, an effect of 25(OH)D_3_ could be detected (32). This is in good agreement with our observations. We found that mono-cultures of activated T cells and activated M2 macrophages could not convert 25(OH)D_3_ to 1,25(OH)_2_D_3_ in the presence of 1 µM DBP. In contrast, mono-cultures of activated M1 macrophages, which expressed ∼10 fold higher levels of CYP27B1 than activated M2 macrophages, did convert significant amounts of 25(OH)D_3_ to 1,25(OH)_2_D_3_ even in the presence of 1 µM DBP. We found that the production of 1,25(OH)_2_D_3_ in co-cultures of activated M1 macrophages and T cells was sufficiently high to affect T cell effector function even in the presence of 1 µM DBP. Thus, the 25(OH)D_3_ to 1,25(OH)_2_D_3_ conversion was sufficiently efficient to allow for 1,25(OH)_2_D_3_-mediated inhibition of IFN-γ and stimulation of IL-5 and IL-13 production in the presence of 1 µM DBP. The capacity to produce sufficient 1,25(OH)_2_D_3_ to affect T cell effector function was further supported by the observation that 1,25(OH)_2_D_3_-mediated regulation of the transcription factors Tbx21, GATA3 and FoxP3 was more efficient in T cell/M1 macrophage co-cultures than in T cell/M2 macrophage co-cultures in the presence of DBP.

Previous studies have indicated that high amounts of 1,25(OH)_2_D_3_ can be produced by granulomas in sarcoidosis, tuberculosis and other granulomatous disorders (33–36). Our study supports these studies and indicates that it is mainly granulomas dominated by M1 macrophages and Th1 cells that produce these high amounts of 1,25(OH)_2_D_3_. These kind of granulomas can cause severe tissue damage and it may be suggested that the production of 1,25(OH)_2_D_3_ is part of a negative feed-back mechanism to reduce tissue damage, as 1,25(OH)_2_D_3_ both inhibits IFN-γ and IL-17A production and induces a shift from M1 towards M2 macrophages (13–15, 43, 44).

## Data Availability Statement

The original contributions presented in the study are included in the article/**Supplementary Material**. Further inquiries can be directed to the corresponding author.

## Ethics Statement

The studies involving human participants were reviewed and approved by Regional Ethical Committee of the Capital Region of Denmark. The patients/participants provided their written informed consent to participate in this study.

## Author Contributions

CG, MK-W, and DL. conceived the study and designed experiments. MK-W, DL, and FA performed the experiments. AW, NØ, and CB. assisted with the experimental design and data interpretation. CG, DL, and MK-W analysed the data and wrote the manuscript with input from all authors. All authors contributed to the article and approved the submitted version.

## Funding

This study was supported by The LEO Foundation grant number LF17058 and The Danish Council for Independent Research grant number 8020-00066B.

## Conflict of Interest

The authors declare that the research was conducted in the absence of any commercial or financial relationships that could be construed as a potential conflict of interest.

## Publisher’s Note

All claims expressed in this article are solely those of the authors and do not necessarily represent those of their affiliated organizations, or those of the publisher, the editors and the reviewers. Any product that may be evaluated in this article, or claim that may be made by its manufacturer, is not guaranteed or endorsed by the publisher.
