# Supplementary material for: Macrophages Control the Bioavailability of Vitamin D and Vitamin D-Regulated T Cell Responses
Source: Front Immunol. 2021 Sep 21;12:722806. doi: 10.3389/fimmu.2021.722806 (PMC8490813; doi:10.3389/fimmu.2021.722806)
Supplement: Supplementary file 1 [file Image_1.pdf]

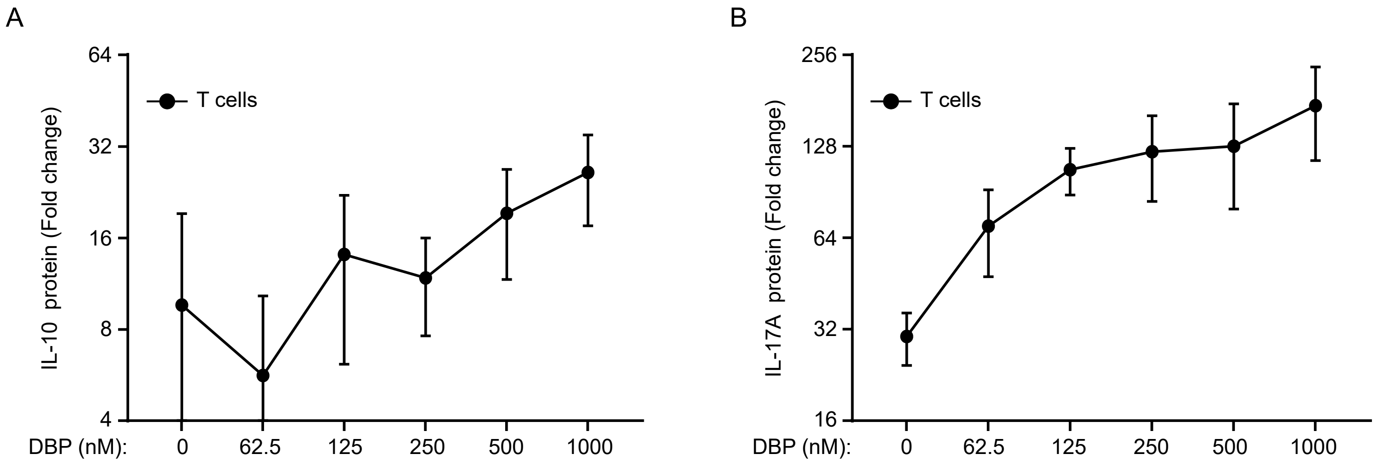

Relative **(A)** IL-10 and **(B)** IL-17A concentrations in the supernatant of $CD4^+$ T cells activated in mono-cultures with Dynabeads Human T-activator CD3/CD28. Each series of data was normalized to the cytokine production in the presence of 100 nM $25(OH)D_3$ and absence of DBP. The data sets were tested using a one-way ANOVA with post hoc multiple comparisons test (Dunnett's) to the cell cultures without DBP.
